# Directional migration of Mg^2+^ in hexagonal Se cathode to unlock high-energy-density Mg metal batteries

**DOI:** 10.1093/nsr/nwaf485

**Published:** 2025-11-07

**Authors:** Kewei Wang, Tongmin Xu, Jie Zhu, Yuming Chai, Yijiang Bao, Shiqiang Feng, Chengkai Yang, Shiyang Wang, Qi Li, Dongsheng Xu

**Affiliations:** Beijing National Laboratory for Molecular Sciences, College of Chemistry and Molecular Engineering; Peking University, Beijing 100871, China; Key Laboratory of Advanced Materials Technologies International (Hong Kong, Macao and Taiwan) Joint Laboratory on Advanced Materials Technologies, College of Materials Science and Engineering, Fuzhou University, Fuzhou 350108, China; Beijing National Laboratory for Molecular Sciences, College of Chemistry and Molecular Engineering; Peking University, Beijing 100871, China; Beijing National Laboratory for Molecular Sciences, College of Chemistry and Molecular Engineering; Peking University, Beijing 100871, China; Beijing National Laboratory for Molecular Sciences, College of Chemistry and Molecular Engineering; Peking University, Beijing 100871, China; Fujian Science & Technology Innovation Laboratory for Optoelectronic Information of China, Fuzhou 350108, China; State Key Laboratory of Structural Chemistry, Fujian Institute of Research on the Structure of Matter, Chinese Academy of Sciences, Fuzhou 350002, China; Key Laboratory of Advanced Materials Technologies International (Hong Kong, Macao and Taiwan) Joint Laboratory on Advanced Materials Technologies, College of Materials Science and Engineering, Fuzhou University, Fuzhou 350108, China; Beijing National Laboratory for Molecular Sciences, College of Chemistry and Molecular Engineering; Peking University, Beijing 100871, China; Beijing National Laboratory for Molecular Sciences, College of Chemistry and Molecular Engineering; Peking University, Beijing 100871, China; Beijing National Laboratory for Molecular Sciences, College of Chemistry and Molecular Engineering; Peking University, Beijing 100871, China

**Keywords:** Mg metal batteries, energy density, directional migration, pouch cell

## Abstract

Conversion-type cathodes have raised much attention in rechargeable Mg metal batteries owing to the low reaction energy barriers and high specific capacities. However, the structural collapse during the charging/discharging cycles often leads to increased electrochemical polarization and capacity degradation. In this work, we demonstrate a directional Mg^2+^ migration strategy in hexagonal selenium (H-Se) cathodes with 3D interconnected chain structures, enabling the sequential diffusion of Cu and Mg ions along the specific crystal plane and the lattice-matching phase conversions from H-Se (100) to Cu_2−x_Se (220) and eventually to MgSe (220). As a result, H-Se exhibits both a high specific capacity (630 mAh g^−1^), excellent rate performance (434 mAh g^−1^ at 2 C), high specific capacity and areal capacity (>500 mAh g^−1^ and 5 mAh cm^−2^) and long lifespan (∼1000 cycles). Finally, a H-Se based prototype pouch cell with a gravimetric energy density of 50 Wh kg^−1^ is achieved.

## Teaser

A Mg^2+^ directional migration strategy is reported in hexagonal selenium via a Cu-catalyzed lattice-matching transformation, which minimizes structural strain and promotes the Mg^2+^ diffusion and lowering the activation energy barrier during the conversion from H-Se to Cu_2−x_Se and finally to MgSe. As a result, a Mg metal pouch cell with a gravimetric energy density of 50 Wh kg^−1^ is achieved.

## INTRODUCTION

Mg metal batteries (MMBs) have emerged as a promising alternative to lithium-ion systems [1–5], driven by the advantages of the Mg metal anode with high crustal abundance (Mg: 2.1% vs. Li: 0.0007%), superior volumetric capacity (Mg: 3833 mAh cm^−3^ vs. Li: 2061 mAh cm^−3^), elevated melting point (Mg: 650°C vs. Li: 180.5°C) and the intrinsic resistance to dendritic growth [6–11]. However, the high chemical activity and low electrode potential (−2.37 V vs. Standard Hydrogen Electrode) of the Mg metal cause the electrolyte decomposition and passivation layer formation at the anode interface [12–14]. Fortunately, these interfacial challenges can be partially solved by advanced electrolyte design, enabling reversible Mg plating/stripping with Coulombic efficiencies exceeding 99% [15–20]. Current limitations of MMBs primarily originate from the lack of suitable cathode materials. Conventional cathodes (e.g. LiCoO_2_, LiFePO_4_) [21–24] in lithium-ion batteries fail to store Mg^2^⁺ due to its strong Coulombic interactions with host lattices and inherently restricted solid-state diffusion. It is essential to develop fundamentally new cathode structures with optimized Mg^2+^ transport pathways to address electrostatic polarization and kinetic limitations [25–27].

To weaken this Mg^2+^–host lattice interaction [28–30], strategies such as solvent co-insertion [15,31], oxygen vacancy engineering [32–34], crystal water [35,36] and interlayer spacing expansion [37,38] have been extensively explored. For example, Hou *et al.* reported a layered Mg_0.15_MnO_2_ cathode with a solvent–Mg^2+^ co-insertion mechanism, which successfully enabled high operation voltage platform over 2.0 V vs. Mg/Mg^2+^ and a high specific capacity of ∼180 mAh g^−1^ in MMBs [15]. Although improving the Mg^2+^ mobility by shield effects, the strategy would increase the amount of electrolytes. An alternative approach replaces rigid O^2−^ frameworks with softer chalcogenide anions. 2D sulfides such as MoS_2_ and TiS_2_ exploit weak interlayer van der Waals interactions to facilitate rapid Mg^2+^ diffusion, yet exhibiting low operating voltage (∼1 V vs. Mg/Mg^2+^) and retaining the inherent low specific capacities characteristic of insertion-type cathodes [39–42].

Conversion-type cathodes, including sulfur [43] and transition metal chalcogenides (e.g. CuS [44], FeS_2_ [45], CuSe [46]), offer a distinct mechanism that bypasses Coulombic limitations entirely. By storing Mg^2+^ through phase transformation reactions rather than intercalation, these materials achieve high theoretical capacities, reduced energy barriers and enhanced reaction kinetics. Nonetheless, the conversion-type cathodes would experience serious structural degradation and volume expansion, causing the pulverization, poor electrical contact, increased parasitic reactions, rapid capacity decay and kinetics limits [47,48]. Thus, the achievement of long-term cyclability and high energy density in MMB cathodes remains unfulfilled, promoting the development of innovative material architectures and mechanisms.

Herein, a directional migration–conversion strategy was developed to circumvent structural degradation and enhance conversion kinetics in hexagonal selenium (H-Se) cathodes. Combined experimental and computational analyses reveal that Cu and Mg ions diffuse along the (100) lattice of the H-Se in sequence. It is found that Se–Se interatomic distances of the subsequent diffusion channels from H-Se (100) (4.37 Å) to Cu_2−x_Se (220) (4.06 Å) and finally MgSe (220) (3.86 Å) reveal that this lattice-matching phase conversion reduces the lattice distortion, promoting the electrochemical kinetics and preserving crystallographic structures of the H-Se. Hence, the H-Se cathode achieves a specific capacity of 630 mAh g^−1^, characteristic of the high capacities of conversion-type cathodes, while overcoming its traditional limitations through achieving high areal capacities, excellent rate performance and long lifespans. The prototype pouch cell demonstrates stable cycling over 75 cycles at a mass loading of 1.6 mg cm^−2^, while achieving an industrially relevant high loading of 4.7 mg cm^−2^ that yields a gravimetric energy density of 50 Wh kg^−1^, which is the highest reported value among MMBs to date.

## RESULTS AND DISCUSSION

### Electrochemical performance of H-Se cathode

**Figure 1.**
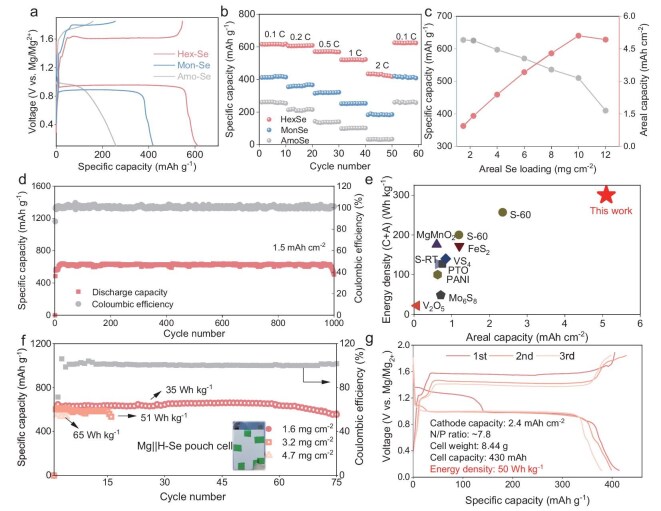
Electrochemical characterizations of the H-Se cathode. (a) First-cycle voltage–capacity profiles of H-Se, M-Se and A-Se at 0.1 C. (b) Rate performance of the three cathode materials. (c) Specific capacity and areal capacity as a function of the mass loading of H-Se. (d) Long-term cycling stability of the H-Se with the areal capacity of 1.5 mAh cm^−^^2^. (e) Comparison of the areal capacities and theoretical gravimetric energy densities of the H-Se cathode with other cathodes from literature. (f) Electrochemical performance of Mg||H-Se pouch cell with different cathode mass loadings. (g) Voltage–capacity curves of the Mg||H-Se pouch cell with a gravimetric energy density of 50 Wh kg^−^^1^.

Three types of Se cathode materials, including H-Se, monoclinic Se (M-Se) and amorphous Se (A-Se) were synthesized via a thermo-melt infusion method (Fig. S1) [49]. Their crystal structures are confirmed by X-ray diffraction (XRD) and transmission electron microscopy (TEM) analyses (Figs S2–S7). Se cathode materials show excellent chemical stability with a Cu current collector (Figs S8–S10). Figure 1a gives the first-cycle galvanostatic charge–discharge curves of the three types of Se cathodes at 0.1 C. Notably, H-Se has a specific capacity exceeding 600 mAh g^−1^, which is higher than that of M-Se (418 mAh g^−1^) and A-Se (255 mAh g^−1^). Notably, the H-Se cathode quickly achieves near-theoretical capacity without exhibiting the overlong activation cycles of traditional conversion-type cathodes, indicating that it is kinetically favorable for Mg^2+^ storage (Figs S11 and S12). The H-Se cathode also possesses an excellent rate performance with a specific capacity of 434 mAh g^−1^ at 2 C (Fig. 1b), in contrast to that of 182 and 30 mAh g^−1^ for M-Se and A-Se, respectively. Figure 1c shows the specific capacity and areal capacity of H-Se as a function of the Se mass loading, which indicates a specific capacity of 500 mAh g^−1^ at a high Se loading of 10 mg cm^−2^ (5 mAh cm^−2^). Interestingly, the H-Se cathode with areal capacity of 1.5 mAh cm^−2^ maintained near-100% capacity retention (∼1000 cycles) at 400 mA g^−1^ (Fig. 1d), demonstrating exceptional structural integrity. Moreover, even under the areal capacity of 4 mAh cm^−2^, the H-Se cathode retains a high specific capacity and excellent electrochemical kinetics (Fig. S13). Comparative analysis on the areal capacities and gravimetric energy density positions H-Se as a frontrunner against existing literature (Fig. 1e, Tables S1 and S2).

The practical viability of H-Se cathodes was evaluated through Mg||H-Se pouch cell configurations. At a mass loading of 1.6 mg cm^−2^, the cell achieves stable cycling over 75 cycles with an areal capacity of ∼1 mAh cm^−2^ (Fig. 1f and Table S3). Scaling to industrially relevant loading (4.7 mg cm^−2^) yielded a total capacity of 0.425 Ah in an 8.44 g full cell, corresponding to a gravimetric energy density of 50 Wh kg^−1^ (Fig. 1g and Fig. S14), which surpasses those of the reported Mg metal pouch cells [16,50]. Though a short circuit occurred after 3 cycles, this competitive energy performance highlights the potential of the H-Se cathode for practical MMBs.

### Electrochemical transformation process of H-Se

**Figure 2.**
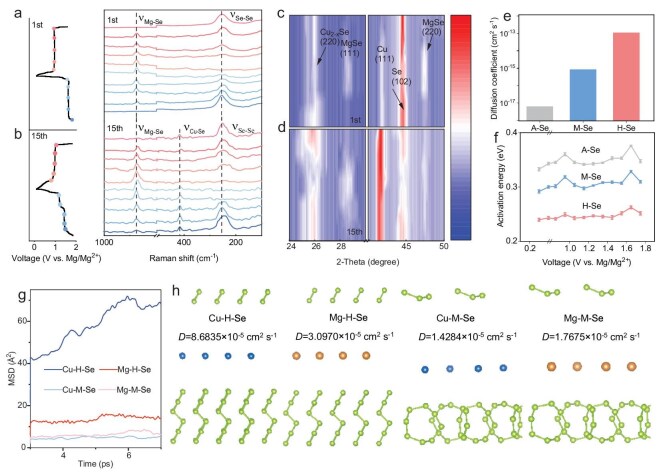
Characterization of Mg storage mechanism in the H-Se cathode. *In situ* Raman characterizations of H-Se cathode with the (a) 1st and (b) 15th cycle. *In situ* XRD characterizations of H-Se cathode with the (c) 1st and (d) 15th cycle. (e) Diffusion coefficient of H-Se, M-Se and A-Se cathode. (f) *E*_a_ of H-Se, M-Se and A-Se cathode. (g) MSD plot and (h) corresponding model of Mg^2^^+^ and Cu^2^^+^ diffusion on H-Se and M-Se.

*In situ* Raman spectroscopy was conducted to investigate the evolution of chemical bonding during galvanostatic cycling, as shown in Fig. 2a and b. During the discharge process (1.85 to 0.1 V vs. Mg/Mg^2+^), the characteristic peaks at ∼260 cm^−1^ associated with the ν_Se–Se_ stretching vibration progressively diminished and eventually vanished, while the ν_Mg–Se_ vibrational mode at 880 cm^−1^ exhibited a concomitant intensity enhancement [46,51]. This trend was reversed during the subsequent charging process. The reversible spectral evolution provides direct spectroscopic evidence for the interconversion between MgSe and H-Se species during electrochemical cycling. Meanwhile, a new Raman peak at ∼480 cm^−1^ originated to the ν_Cu–Se_ vibrational mode would increase with the cycling, suggesting the electrochemically activated involvement of copper species in the redox process (Fig. S15). The reversible conversion of H-Se and cubic MgSe can also be observed by the *in situ* XRD analysis, where the peaks corresponding to the (102) plane of H-Se and (220) plane of cubic MgSe vary inversely (Fig. 2c and d). The involvement of copper species is in the form of the cubic Cu_2−x_Se, evidenced by the appearance of the (220) plane of cubic Cu_2−x_Se, along with the exclusive compatibility observed with the copper current collector during electrochemical characterizations (Fig. S16). We have noticed that the (102) plane of H-Se remains after 5 cycles, demonstrating the crystallographic integrity of H-Se during the cycles.

Figure 2e and f gives kinetic information of the H-Se cathode by the complementary galvanostatic intermittent titration technique (GITT) coupled with electrochemical impedance spectroscopy (EIS). Quantitative analysis of Mg^2+^ diffusivity revealed an order-of-magnitude enhancement in H-Se, exhibiting a Mg^2+^ diffusion coefficient *(D)* of 1.07 × 10^−13^ vs. 8.27 × 10^−16^ cm^2^ s^−1^ for M-Se and 6.21 × 10^−18^ cm^2^ s^−1^ for A-Se (Figs S17–S19). The kinetic advantage was further corroborated by activation energy (*E*_a_) profiling across charge/discharge states, indicating significant reductions in *E*_a_ of H-Se compared to M-Se and A-Se (Figs S20–S22). In addition, the transition in *E*_a_ observed at ∼0.9 V and 1.7 V, which might be attributed to the transformation of Se to MgSe and the reverse transformation of MgSe back to Se, respectively. These observations collectively validate their kinetics differences between H-Se and M-Se or A-Se.

Mean squared displacement (MSD) analysis was employed to assess the diffusion kinetics of Cu and Mg ions in H-Se and M-Se matrices. As demonstrated in Fig. 2g and h, H-Se exhibited superior ion diffusion characteristics relative to M-Se, with calculated MSD values for both Cu (8.68 × 10^−5^ cm^2^ s^−1^) and Mg (3.10 × 10^−5^ cm^2^ s^−1^) exceeding those in M-Se by ∼6- and 1.8-fold, respectively. This enhanced mobility directly correlates with the 3D interconnected chain architecture of H-Se, which facilitates low-energy-barrier ion migration pathways. In contrast, M-Se with isolated Se_8_ ring motifs lacks 3D connected pathways for ion transport.

### Mg and Cu ion migration in H-Se

**Figure 3.**
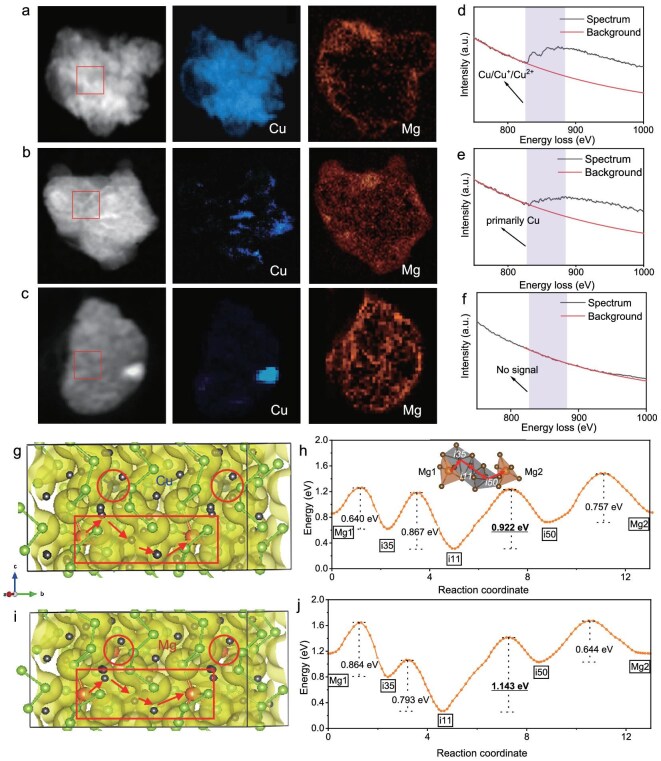
Mg and Cu ion diffusion in the H-Se cathode. STEM images and elemental mapping of the H-Se cathode with (a) 25%, (b) 50% and (c) 75% discharging state. (d–f) Corresponding energy loss spectra of the red box of the H-Se cathode with different discharging states. Crystal structures of (g) Cu@H-Se and (i) Mg@H-Se with the Mg-ion potential mapping (yellow) marked and tracing low-energy interstitial migration sites (black spheres) from BVSE simulations. Energy profiles of the migration pathways in (h) Cu@H-Se and (j) Mg@H-Se. Illustration figure in (h) is the I-interstice leap migration process (Mg1–i35–i11–i50–Mg2) of Cu@H-Se and Mg@H-Se.

Comprehensive microstructural analysis via TEM reveals distinct structural evolution pathways for H-Se versus M-Se and A-Se cathodes. H-Se maintains crystallographic integrity with stabilized grain sizes (∼30 nm, Figs S23–S25), while M-Se and A-Se undergo severe amorphization and particle fragmentation (Figs S26 and S27). EDS mappings elucidate cation diffusion processes in the H-Se cathode: initial Cu-dominated bulk diffusion forms Cu_2−x_Se intermediates (25% discharge, Fig. 3a), followed by Mg^2+^ infiltration displacing Cu ions into interfacial regions (50% discharge, Fig. 3b), and finally in metallic Cu nanoparticle extrusion (75% discharge, Fig. 3c). Concurrent electron energy loss spectroscopy (EELS) proves that the valence of Cu transition from mixed oxidation states (Cu^2+^/Cu^+^/Cu^0^) at 25% discharges to predominantly metallic Cu^0^ at 50% discharge and the complete disappearance of Cu signals in bulk regions at 75% discharge (Fig. 3d–f). In addition, we also observed the generation of Cu nanowires in long-cycled cathodes, which can be attributed to the directional Cu diffusion and the subsequent nucleation and growth (Fig. S28).

Bond valence site energy (BVSE) calculation was conducted to reveal the critical role of Cu ions in influencing the Mg^2+^ diffusion kinetics in H-Se cathodes. Fig. 3g and i presents optimized structural models of Cu- and Mg-incorporated H-Se frameworks (denoted Cu@H-Se and Mg@H-Se, respectively). The Mg^2+^ migration pathways originating from the red dashed boxes of Fig. 3g and i prove that the Mg^2+^ diffused along the same direction and migration path in the two structural models. Figure 3h and j displays the Mg^2+^ migration energy variations in Cu@H-Se and Mg@H-Se frameworks, respectively, which identifies the reduced rate-determining step (0.92 eV) in Cu@H-Se compared to that in Mg@H-Se (1.14 eV) from *i_11_* to *i_50_*. Such a 19.3% kinetics enhancement may be attributed to the Cu-induced lattice matching and charge shielding at Mg coordination sites, demonstrating the diffusion of Cu in facilitating Mg^2+^ migration kinetics.

### Mg storage mechanism of H-Se

**Figure 4.**
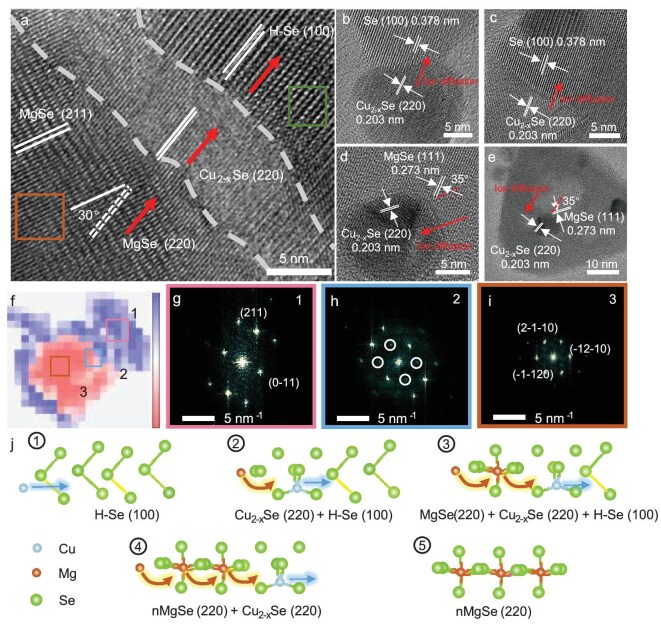
Structural evolution of the H-Se cathode. (a) TEM images of the discharged H-Se cathode. (b and c) The interface between H-Se (100) and Cu_2__−__x_Se (220). (d and e) The interface between Cu_2__−__x_Se (220) and MgSe (220). (f) SEND map of the H-Se and diffraction patterns of (g) the unreacted Se, (h) the intermediate phase and (i) the converted MgSe. (j) Schematic diagram of the conversion from H-Se to cubic MgSe with the Cu-catalyzed process.

TEM analysis confirms the structural evolution of cycled H-Se cathodes, revealing coherent three-phase interfaces between H-Se (*d*_100_ = 0.378 nm), cubic Cu_2−x_Se (*d*_220_ = 0.203 nm) and cubic MgSe (220) via lattice-fringe measurements, fast Fourier transform (FFT) pattern indexing and lattice rotation (Fig. 4a and Figs S29–S31). It is notable that the (220) plane of cubic MgSe can be observed by rotating the (211) plane by 30°, which aligns well with Cu_2−x_Se (220), as the dotted line shows (Fig. S32). Other crystallographic images also indicate the lattice-matching transformation mechanism by the coherent alignment of H-Se (100), cubic Cu_2−x_Se (220) and MgSe (220) (Fig. 4b–e and Fig. S33). Scanning electron nano-diffraction (SEND) map and high-angle annular dark-field scanning TEM (HAADF-STEM) analysis also track the structural evolution of the cycled H-Se cathodes, with the existence of H-Se, intermediated cubic Cu_2−x_Se and MgSe during the discharging process (Fig. 4f–i and Fig. S34).

According to the above results, we conclude the Mg^2+^ migrating mechanism as shown in Fig. 4j. Firstly, the H-Se cathode with 3D channels enables rapid Cu diffusion and the conversion of cubic Cu_2−x_Se with its (220) lattice plane aligned to the (100) of H-Se. The Cu ion then further diffuses along the (100) plane of the H-Se to continuously produce the cubic Cu_2−x_Se and leave the tetrahedral vacancy, and subsequently fast Mg^2+^ diffusion and substitution proceeds into the hexahedral vacancy. Finally, all the H-Se structures are converted to cubic MgSe through a vacancy-mediated exchange. Notably, the progressive reduction in Se–Se interatomic distances of the diffusion channels from H-Se (100) (4.37 Å) to Cu_2−x_Se (220) (4.06 Å) and finally MgSe (220) (3.86 Å) reveals the critical role of Cu_2−x_Se intermediates in regulating gradual lattice distortion, thereby minimizing structural strain, promoting the Mg^2+^ diffusion and lowering the *E*_a_ barrier during the conversion from H-Se to MgSe. This directional diffusion and sequential lattice-matching mechanism enables kinetically favorable phase transitions while preserving crystallographic characters of the Se cathode.

## CONCLUSION

In summary, a directional diffusion–conversion strategy was developed in H-Se cathodes to enable high-performance MMBs, in which Mg^2+^ ions diffuse along an H-Se (100) channel via a Cu-catalyzed lattice-matching transformation. The subsequent diffusion–conversion mechanism minimizes lattice strain and reduces reaction energy barriers. As a consequence, the H-Se cathode synergistically combines conversion-type and insertion-type electrochemistry, resulting in a high specific capacity, excellent cycling stability, high rate performance and areal capacity loading. This directional diffusion strategy might provide new insights for the development of multivalent metal batteries.

## Supplementary Material

nwaf485_Supplemental_File

## Data Availability

All data are available in the main text or the Supplementary data.
