# Supplementary material for: Directional migration of Mg2+ in hexagonal Se cathode to unlock high-energy-density Mg metal batteries
Source: Natl Sci Rev. 2025 Nov 7;13(2):nwaf485. doi: 10.1093/nsr/nwaf485 (PMC12860199; doi:10.1093/nsr/nwaf485)
Supplement: nwaf485_Supplemental_File [file nwaf485_supplemental_file.pdf]

Supporting Information

Directional Migration of Mg^{2+} in Hexagonal Se Cathode to Unlock High-Energy-Density Mg Metal Batteries

Kewei Wang[1,†], Tongmin Xu[2,†], Jie Zhu[1], Yuming Chai[1], Yijiang Bao[1], Shiqiang Feng[3,4,*], Chengkai Yang[2,*], Shiyang Wang[1,*], Qi Li[1], Dongsheng Xu[1,*]

[1]Beijng National Laboratory for Molecular Sciences, College of Chemistry and Molecular Engineering; Peking University, Beijing 100871, China

[2]Key Laboratory of Advanced Materials Technologies International (Hong Kong Macao and Taiwan) Joint Laboratory on Advanced Materials Technologies, College of Materials Science and Engineering, Fuzhou University, Fuzhou, Fujian 350108, China

[3]Fujian Science & Technology Innovation Laboratory for Optoelectronic Information of China, Fuzhou 350108, China

[4]State Key Laboratory of Structural Chemistry, Fujian Institute of Research on the Structure of Matter, Chinese Academy of Sciences, Fuzhou 350002, China

*Corresponding author E-mail: dsxu@pku.edu.cn; wangshiy@pku.edu.cn; chengkaiyang@fzu.edu.cn; fsqvictor@pku.edu.cn

[†]These authors contributed equally to this work.

Experimental Section

Materials: Anhydrous $MgCl_2$ (99.9%, Sigma–Aldrich) and $AlCl_3$ (99.999%, Sigma–Aldrich), Carbon black (Super-P, 99%, Sigma–Aldrich), anhydrous tetrahydrofuran (THF, 99.5%, H_2O≤30 ppm, J&K Scientific), anhydrous 1,2-Dimethoxyethane (DME, 99.5%, H_2O≤30 ppm, J&K Scientific), $Mg(TFSI)_2$ (99.5%, Solvionic), Selenium (Se powder, 99%, BEIJING TONG GUANG FINE CHEMICALS COMPANY), polyvinylidene fluoride (PVDF, 99%, Alfa-Aesar), 1-Methyl-2-pyrrolidinone (NMP, 99%, J&K Scientific), all the chemicals were used without further purification.

Synthesis of Se/C: Commercial selenium powder and carbon black (Super-P) were mixed and grounded evenly according to the mass ratio of 7:3 or 6.5:1. The mixture was encapsulated in a porcelain boat and calcined at 260 °C for 6 h, and then slowly cooled to obtain the H-Se/C composite, or rapidly cooled to obtain the A-Se/C composite, or heated to 450 °C and cooled to obtain the M-Se/C composite. H-Se can be purified by carbon disulfide.

Preparation of electrolytes: Preparation of MACT ($MgCl_2/AlCl_3/Mg(TFSI)_2$, 2/1/1) electrolyte (0.25 mol L^{-1} MACT-DME/THF electrolyte): a certain amount of $MgCl_2$ (0.5 mol L^{-1}), $AlCl_3$ (0.25 mol L^{-1}) and $Mg(TFSI)_2$ (0.25 mol L^{-1}) were added into the mixture of DME and THF (v/v=1:1), after stirring for 24 h in an argon-filled glovebox, the hybrid electrolyte was obtained.

Preparation of Se electrodes: For all the experiments in this work unless indicated, Se/C, and PVDF binder (3.2% PVDF in NMP) were mixed in a 9:1 mass ratio, pasted on Copper Foil (cold rolling Cu, one side for coin cell, two side for pouch cell), and dried at 60 °C before use. The mass loading of the active material ranges from 1.5 to 15 mg cm^{-2}.

Electrochemical measurements: All the electrochemical tests, except for those on the Mg metal pouch cells and in situ cells, were conducted by 2032-type coin cells. Mg foil was polished until the surface became shiny before use. For coin cells, glass fiber (GF-D) separator and adequate electrolytes (100 μL) were used for cycling. For pouch cells, Mg foil (100 μm, 50 μm for each side), PVDF separator (100 μm), and

lean electrolytes (E/C<2.5) were used for cycling. Cyclic voltammetry (CV) was tested on a CHI660A electrochemical workstation. Galvanostatic charge–discharge curves were recorded on a LAND electrochemical workstation (Wuhan LAND Electronic Co. Ltd., China).

Structure and conductivity characterizations: The morphologies and structures of the materials were characterized by XRD (PANalytical X-Pert3 Powder), SEM (Hitachi S-4800), and TEM (JEM-2100F). The Se cathodes with different crystal phases were pressed into discs, and the conductivity of the materials was measured directly by a digital source meter. And the conductivity under different pressures was measured by a four probe conductivity meter.

Operando Raman spectroscopy characterization: Operando Raman tests were performed by the DXRxi Raman Microscope. In situ cells (Supplementary Fig. 9, Supporting Information) were conducted by a LAND battery analyzer with simultaneous Raman measurements. A 1 mm thick Quartz glass (10 mm in diameter) was used as a window, and the positive electrode preparation is the same as before (19 mm in diameter), while the GF-D (20 mm in diameter with a hole of 10 mm) was used as separator, Mg foil (19 mm in diameter with a hole of 12 mm) as anode. An incident laser with a wavelength of 532 nm was irradiated through the quartz glass and holes to the positive electrode. Operando Raman measurements were tested every 5 min during the galvanostatic cycling of 0.2 C.

Operando XRD characterization: The powder X-ray diffraction (XRD) patterns were obtained from Bruker D8 Advance diffractometer with a Cu Kα (λ=1.5418 Å) source, and refined using DIFFRAC TOPAS V5 software. The in-situ XRD was carried out with a home-made in suit XRD cell. The assembly process is the same as the coin cell, and a small amount of vacuum silicone was greased on the back of the Cu foil and closely adhered to the opening hole of the in situ cell. X-ray was irradiated through the Cu foil to the back of the positive electrode. Operando XRD measurements were tested every 10 min during the galvanostatic cycling of 0.5 C.

XPS characterization: The valence states of Cu were investigated by XPS (ESCALAB Xi+, Thermo Fisher Scientific). Cycled cathodes washed by DME were

sealed in an airtight transfer container during the transfer process and opened after being loaded onto the stage inside the transfer chamber to prevent the exposure of samples to air. All of the spectra were calibrated with the C-C peaks at 284.8 eV.

TEM characterization: TEM and scanning transmission electron microscopy (STEM) images were performed on the JEM-2100F transmission electron microscope and Themis Z Spherical Aberration-Corrected Transmission Electron Microscopy. The annular dark-field-STEM images and electron energy loss spectroscopy (EELS) spectra were collected using a JEOL-F200 electron microscope (equipped with a Gatan 1077 EELS Spectrometer) operating at a 200 kV accelerating voltage. A cryo-TEM holder (Fischione 2550) was employed to mitigate electron beam-induced sample damage during characterization. Scanning electron nano-diffraction map was conducted by 4D STEM.

STEM-EELS tomographic reconstruction: Tilt series data (-65°, -30°, 0°, 30° and 65°) were acquired with the 42×43 pixels core-loss EELS spectra (from 650 to 2165 eV), featuring 0.75 eV channel dispersion and 0.05 s exposure time. Zero-loss EELS spectra (0 eV energy shift) were recorded under similar conditions with 0.0001 s exposure time. Projection views derived from these spectra enabled 3D structural reconstruction through simultaneous iterative reconstruction technique (SIRT). The SIRT algorithm has been integrated into the Tomopy package and the Astra toolbox in Python, leveraging graphic processing unit (GPU) acceleration for computational efficiency enhancement.

Activation energy calculation: Electrochemical impedance spectroscopy (EIS) was measured at the open circuit and specific voltages under a sinusoidal signal over the frequency range from 0.01 Hz to 100 kHz with an amplitude of 5 mV. The activation energies for various Se conversion steps were obtained from EIS in a Mg||electrolyte||Se full cell based on Arrhenius equation:

$$k=Ae^{\frac{-E_a}{RT}}$$

where k is the rate constant ($1/R_{ct}$, the charge transfer resistance (R_{ct}) was derived from the EIS profiles), T is the absolute temperature, A is the pre-exponential factor,

E_a is the activation energy of the reaction, and R is the universal gas constant.

Mg^{2+} diffusion coefficient determination: Galvanostatic intermittent titration technique (GITT) experiments were performed on LAND battery analyzer. GITT was used to determine the Mg^{2+} diffusion coefficient of Se electrode. The GITT diffusivity can be described by the Equation:

$$D_s=\frac{4}{\pi\tau}\left(\frac{n_M V_M}{S}\right)^2\left(\frac{\Delta E_s}{\Delta E_t}\right)^2$$

where S is the interfacial area, which can be simplified to the surface area of the active material, and τ is the time duration of the pulse, n_M and V_M are respectively the amount of substance (mol) and molar volume (cm^3 mol^{-1}) of the active material, ΔE_s is the steady-state voltage change of the battery after a relaxation period, and ΔE_t is the voltage change of the battery during the time duration of the pulse (τ), with a deduction of the voltage change of ohmic and charge transfer resistances. From the GITT curve, ΔE_s and ΔE_t can be easily identified.

Pouch cell fabrication: The overall device performance of the Mg-Se batteries was evaluated in the pouch cells (4.3 cm × 5.6 cm in dimension). The pouch cell fabrication process was carried out in a dry room by stacking the cathode of Se on Cu current collector, Mg anode and PVDF separator layer by layer, followed by the injection of the electrolyte (E/S ratio = 2 μL mg^{-1}) and vacuum standing for 10 min to remove the excess electrolyte before the final encapsulation in an argon-filled glovebox. The Al and Ni tabs were welded together with the cathodes and anodes, respectively, and introduced for outward connection.

Energy density estimation: The calculation of energy density of cathode material is based on the equation of $E_c = E \times C_a$, where E is the cathode work voltage, C_c is the cathode specific capacity, refering to the calculation of Liu et al.[47] In order to further describe the actual energy density of a material, we calculated the energy density of the battery based on the equation of $E_a = E_c/(m_{cathode} + m_{andoe})$, where $m_{cathode}$ contians active species, binder, conductive materials, etc. and 50 μm Mg foil is used as representative of negative electrode. Finally, the calculation of the energy density of the pouch cell refers to the calculation of Aurbach et al.[1]

Calculation: All spin-polarized density functional theory (DFT) calculations were performed using the Vienna Ab initio Simulation Package (VASP). The exchange-correlation interactions were treated within the framework of the generalized gradient approximation (GGA) using the Perdew-Burke-Ernzerhof (PBE) functional. A plane-wave cutoff energy of 400 eV was applied for the basis set expansion. The electronic self-consistent field (SCF) calculations were considered converged when the total energy change was less than10^{-6} eV. Structural relaxations were performed until the residual forces on each atom were below 0.02 eVA^{-1}. Structural optimizations were performed in the first Brillouin zone using a $1\times2\times1$ Gamma-centered k-point mesh. Bond valence site energy (BVSE) calculations were conducted to visualize the available migration pathways and evaluate the migration barriers within the given structures. Although the migration barriers obtained by the BVSE method are typically higher than those measured experimentally or calculated by other DFT-based methods, BVSE remains a powerful tool for assessing ionic migration behavior. In this study, BVSE calculations were employed to investigate the migration pathways and static migration potentials of Cu@H-Se, and Mg@H-Se structures.

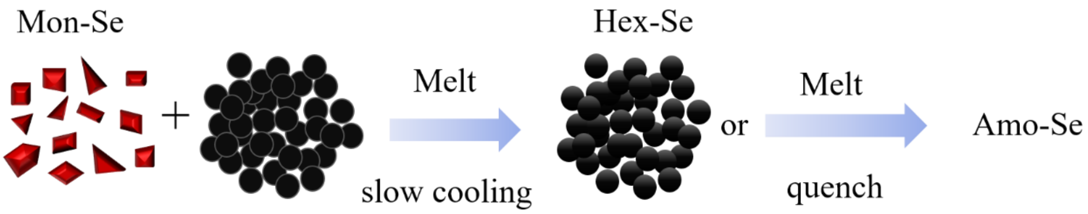

Figure S1. Schematic diagram of the synthesis process of H-Se and A-Se.

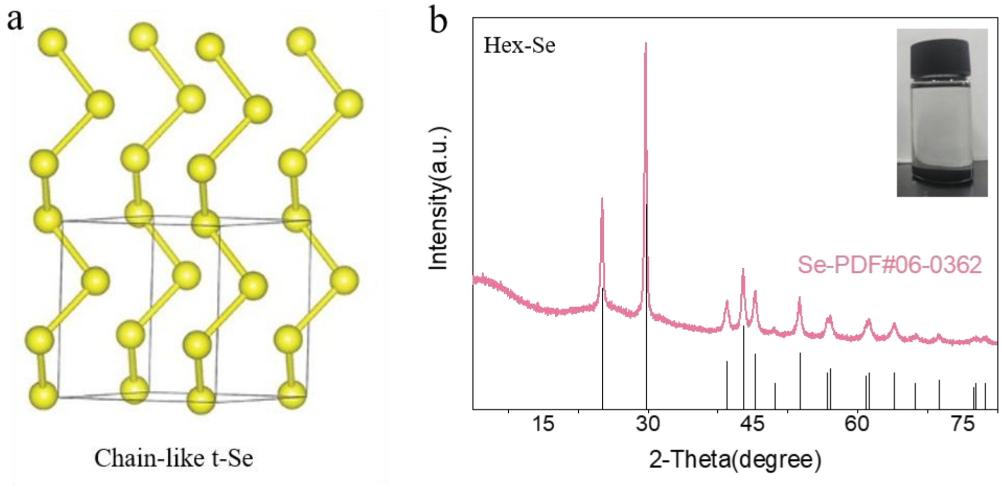

Figure S2. (a) Crystal structure and (b) XRD pattern of the H-Se.

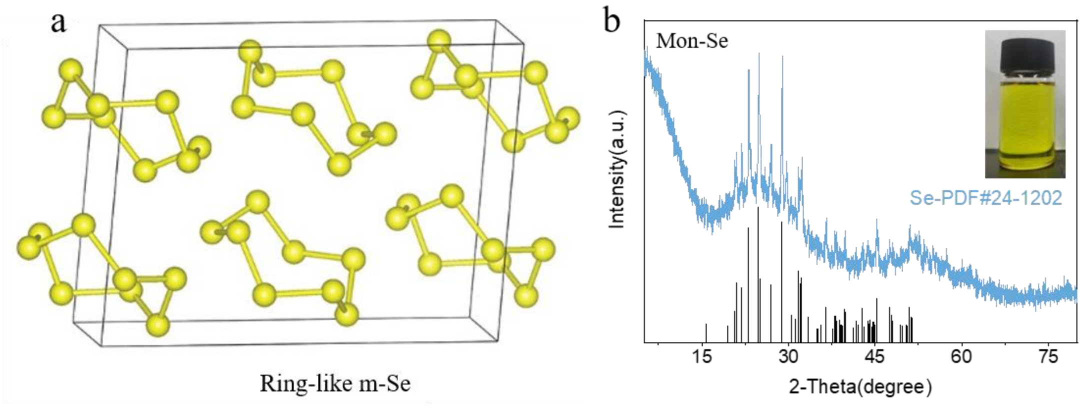

Figure S3. (a) Crystal structure and (b) XRD pattern of the M-Se.

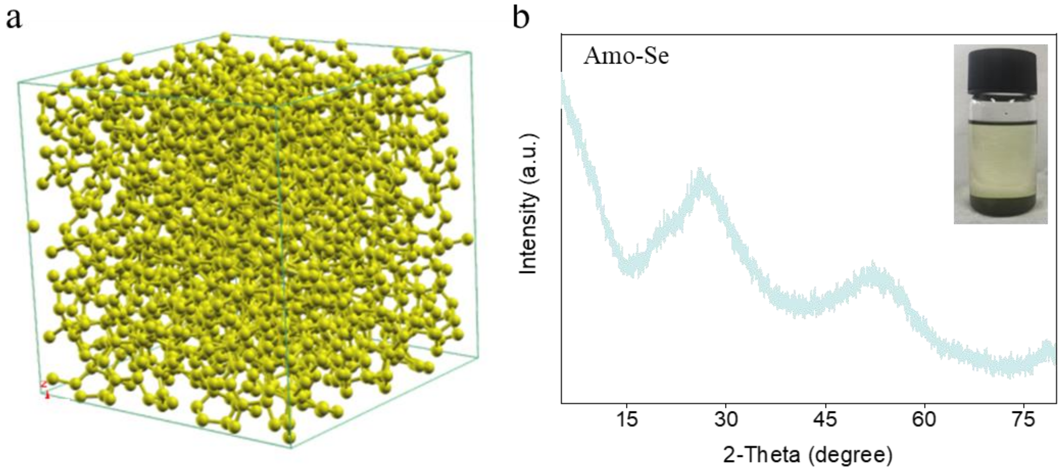

Figure S4. (a) Crystal structure and (b) XRD pattern of the A-Se.

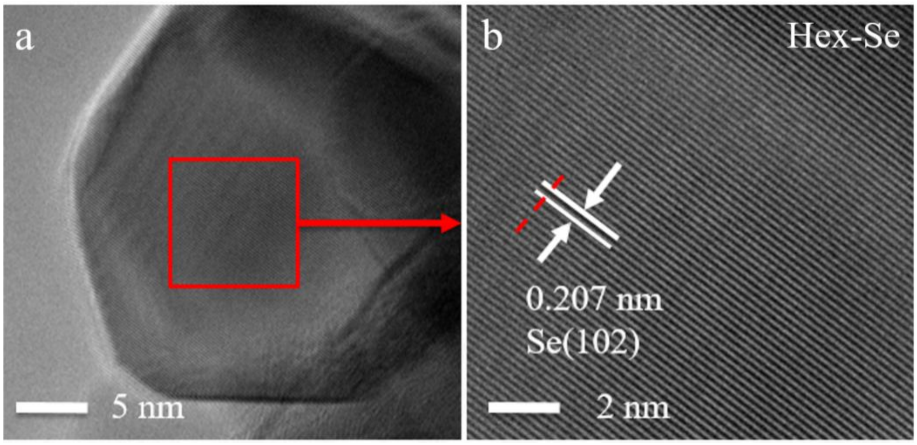

Figure S5. TEM images of (a) H-Se and its (b) lattice stripes.

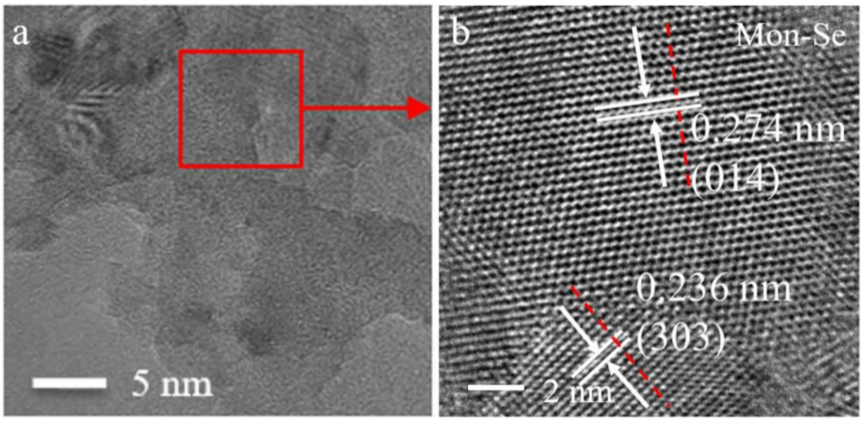

Figure S6. TEM images of (a) M-Se and its (b) lattice stripes.

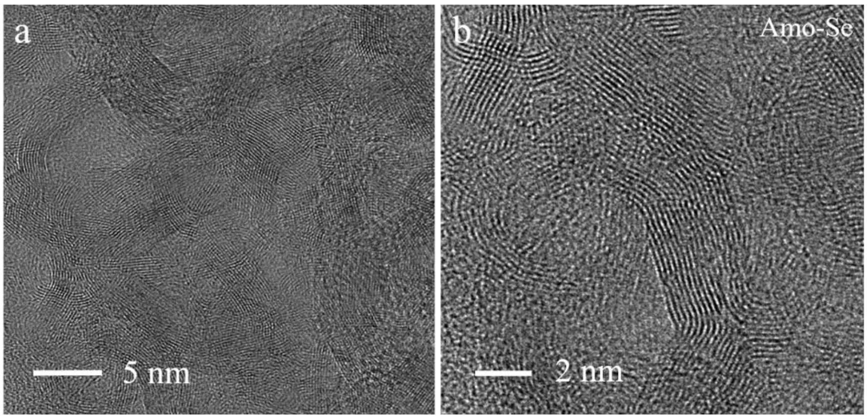

Figure S7. TEM images of (a) Amo-Se and its (b) lattice stripes.

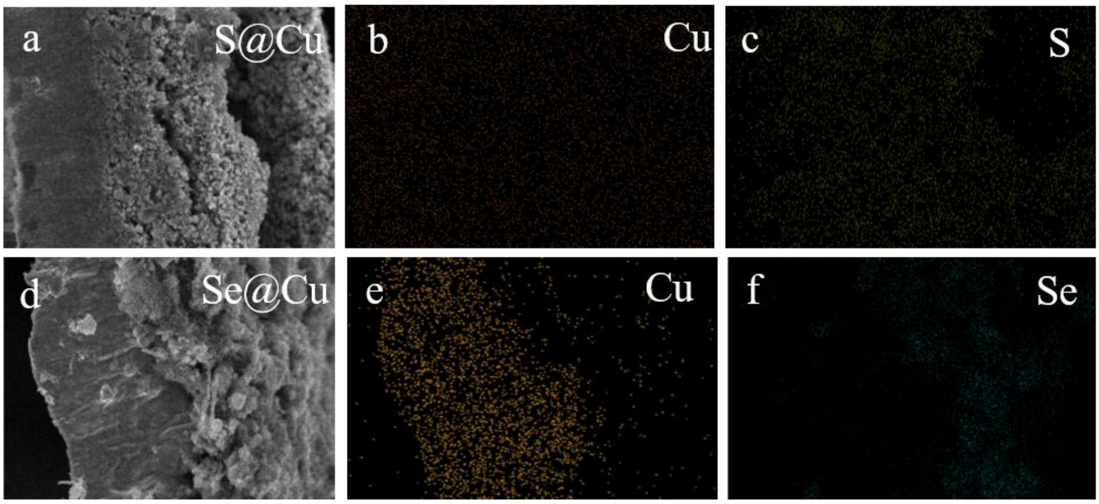

Figure S8. SEM images of (a) S@C-Cu and (d) Se@C-Cu electrodes. The corresponding (b) Cu and (c) S element mapping of S@C-Cu. The corresponding (e) Cu and (f) Se element mapping of Se@C-Cu.

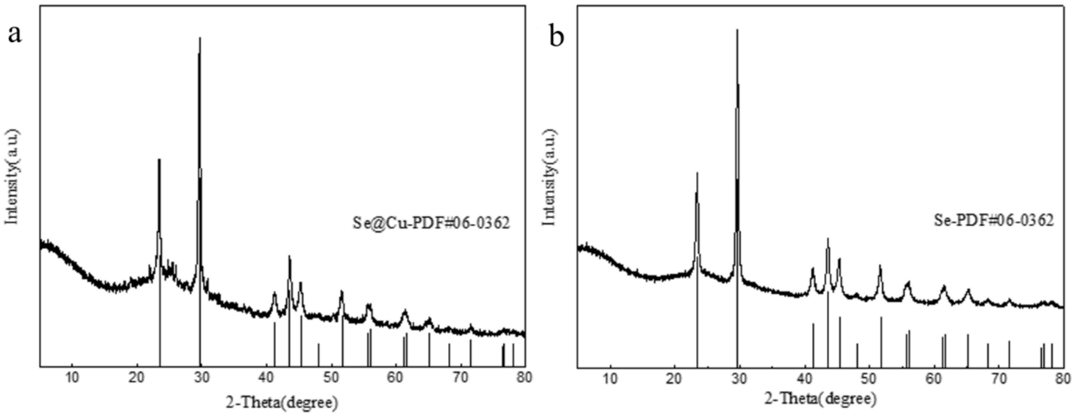

Figure S9. XRD characterizations of Se@C electrode (a) after and (b) before contacting the Cu current collector.

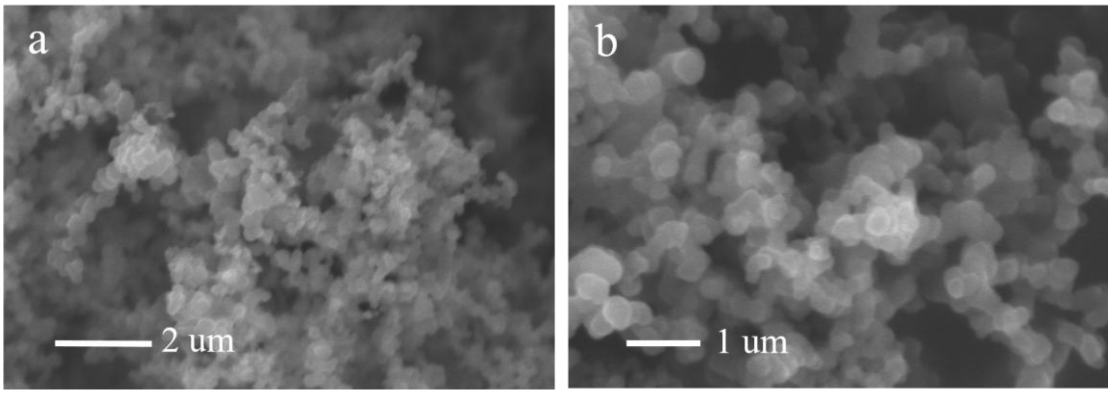

Figure S10. SEM images of Se@C composite with different magnifications.

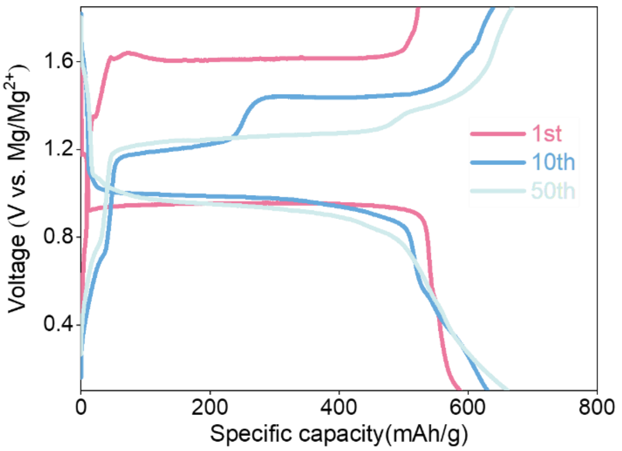

Figure S11. Voltage-capacity profiles of the H-Se||Mg coin cell.

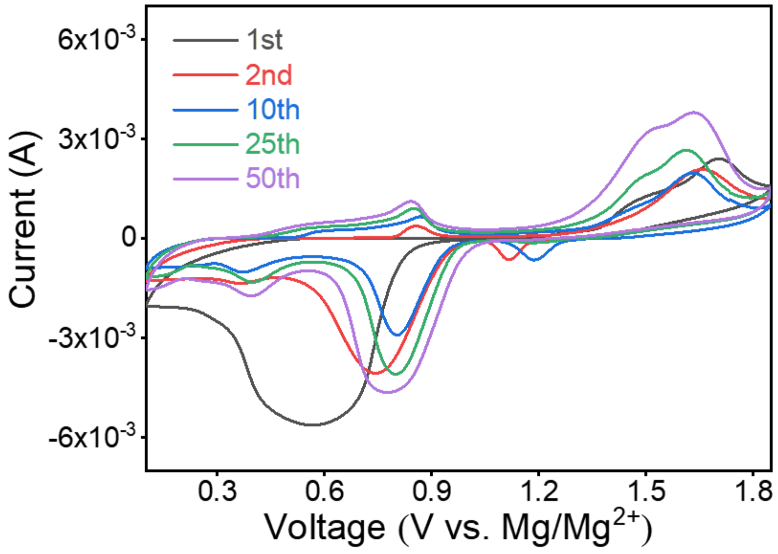

Figure S12. CV characterization of Mg||H-Se cell.

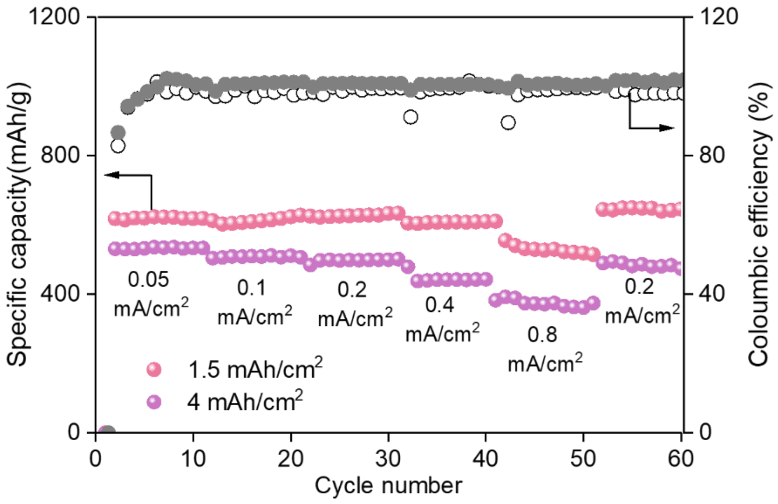

Figure S13. Rate performance of H-Se||Mg coin cell with high areal capacity of 1.5 mAh cm^{-2} and 4 mAh cm^{-2}.

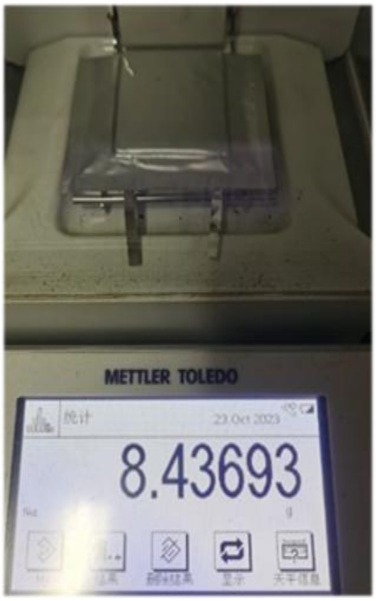

Figure S14. The weight of the pouch cell.

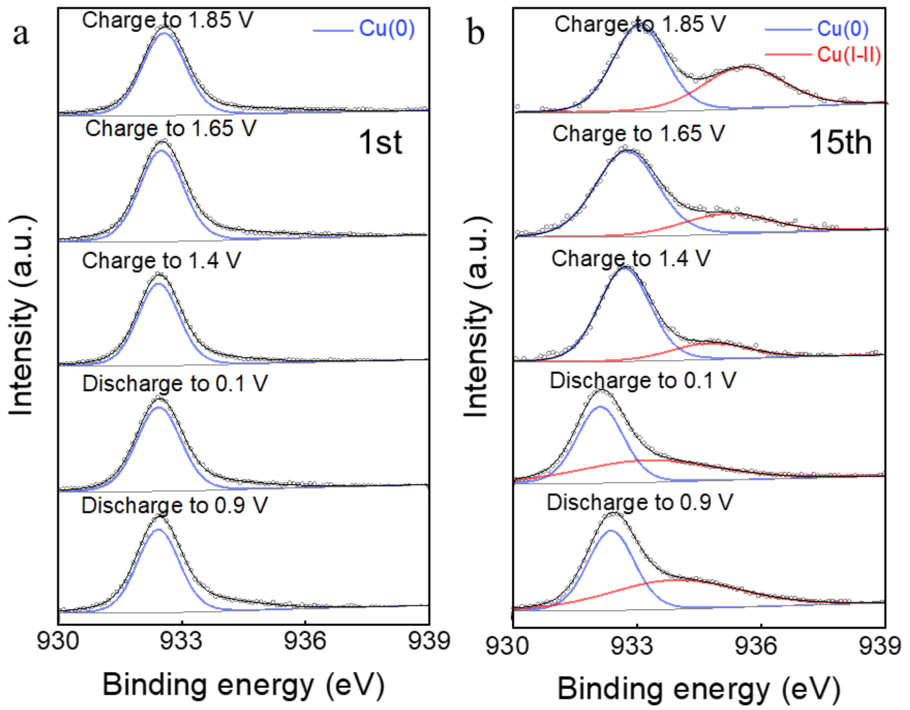

Figure S15. XPS characterization of Cu species during the charging/discharging processes at (a)1st and (b)15th cycles.

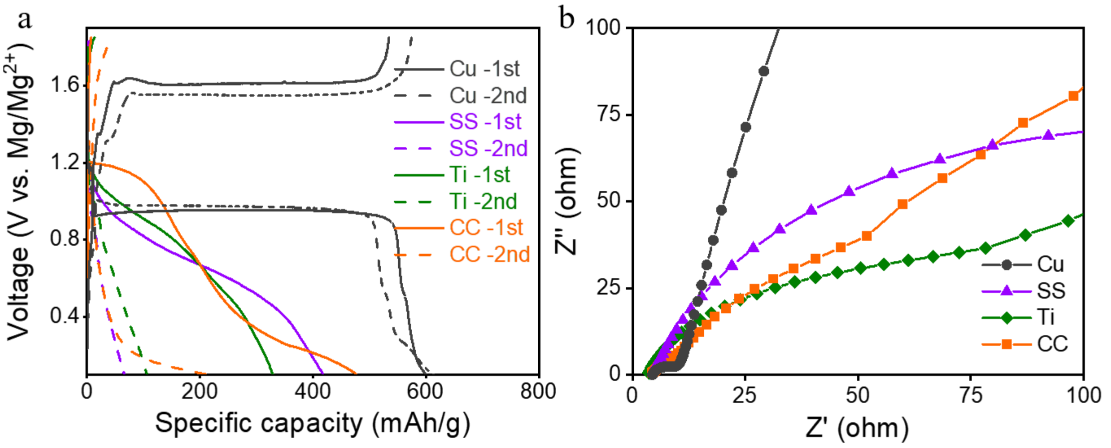

Figure S16. (a) Capacity-voltage profiles and (d) EIS spectra of Mg||Se cell with different current collectors.

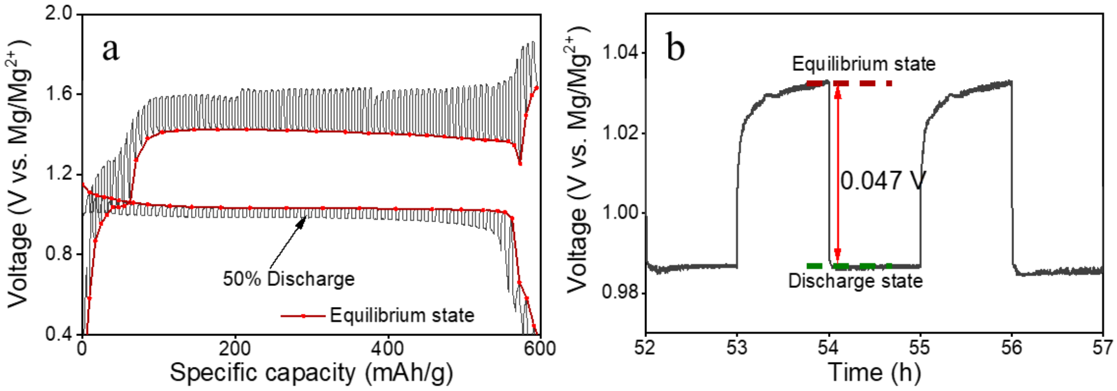

Figure S17. (a) The voltage curves of the Mg||H-Se cell. (b) The corresponding zoomed-in view.

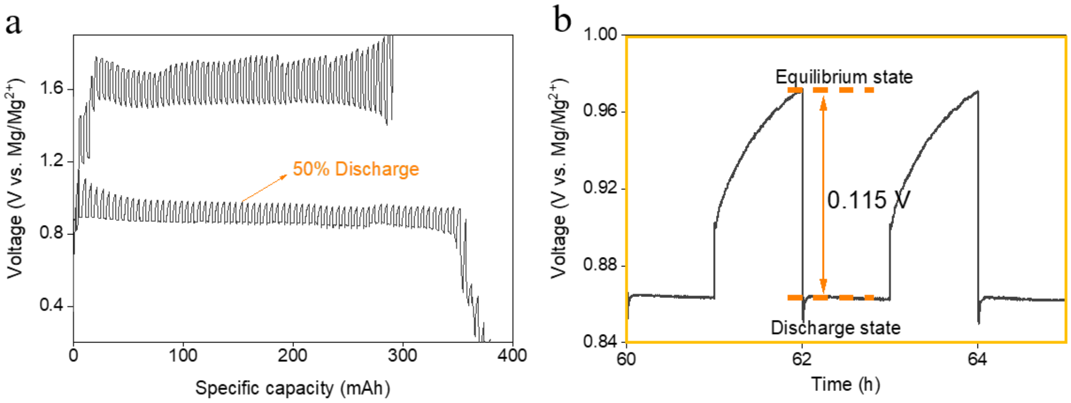

Figure S18. (a) The voltage curves of the Mg||M-Se cell. (b) The corresponding zoomed-in view.

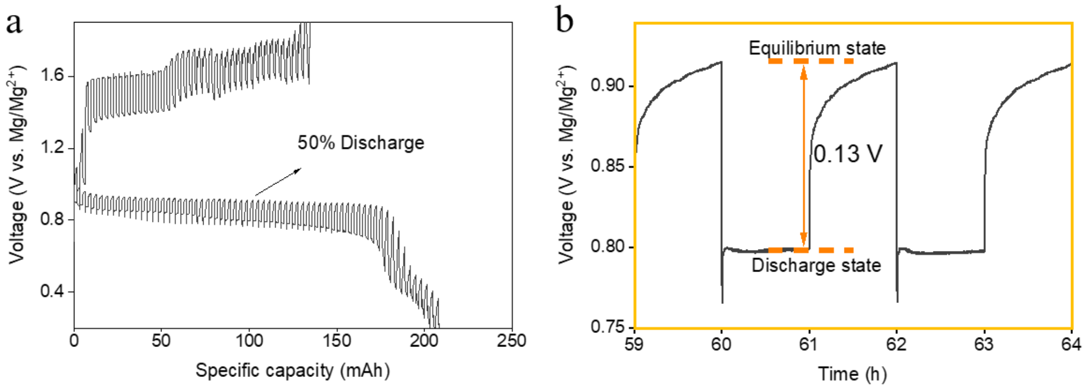

Figure S19. (a) The voltage curves of the Mg||A-Se cell. (b) The corresponding zoomed-in view.

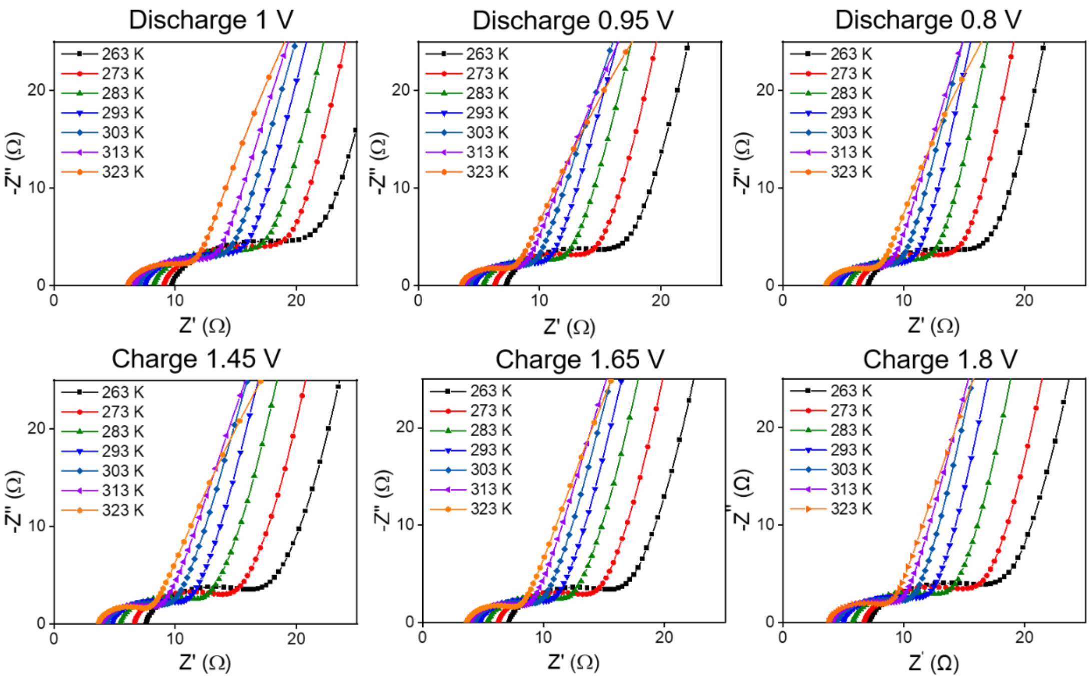

Figure S20. EIS characterizations of Mg||H-Se cell at different temperatures during the charging/discharging processes.

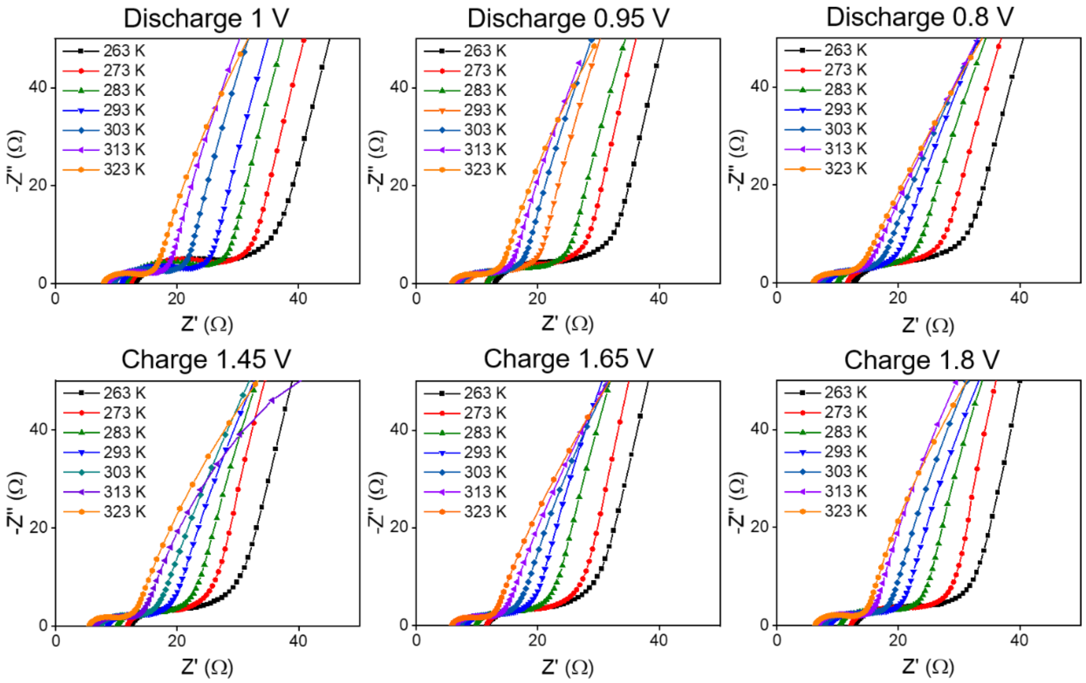

Figure S21. EIS characterizations of Mg||M-Se cell at different temperatures during the charging/discharging processes.

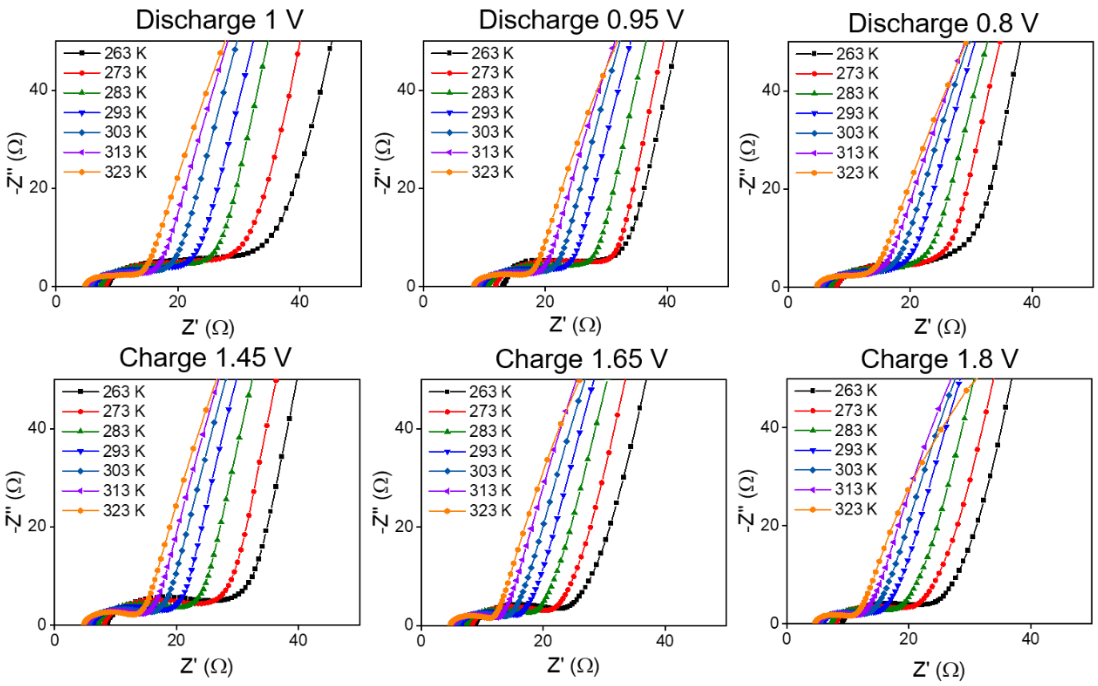

Figure S22. EIS characterizations of Mg||A-Se cell at different temperatures during the charging/discharging processes.

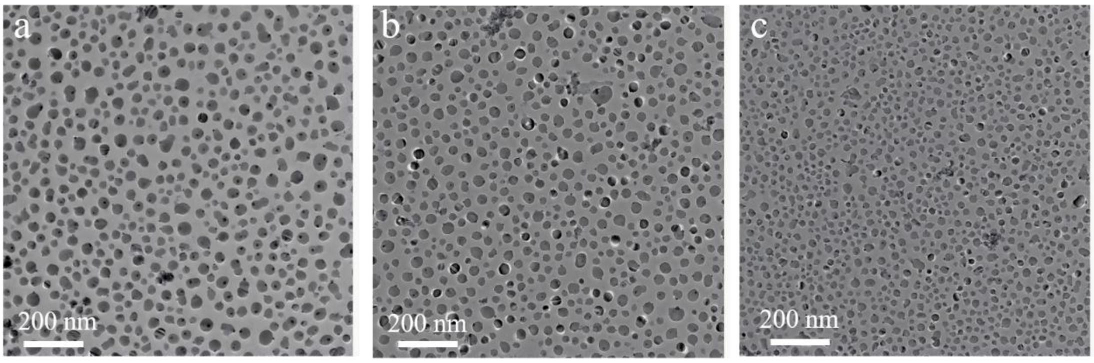

Figure S23. TEM images of the cycled H-Se cathode at (a) 1st, (b) 15th and (c) 40th cycles.

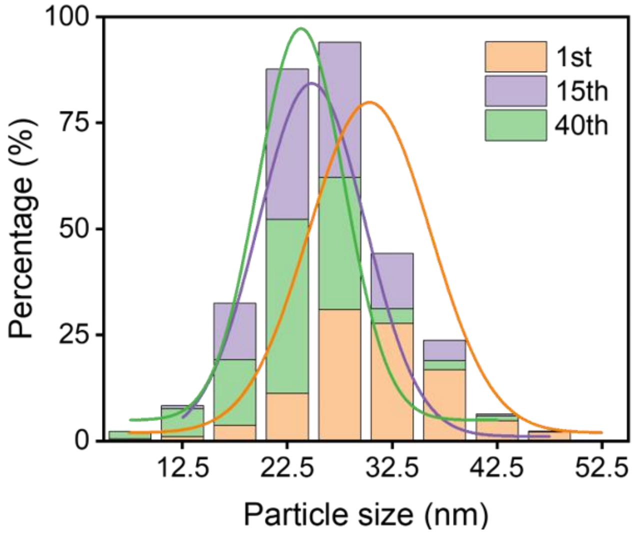

Figure S24. Statistics results of the particle size of the cycled H-Se cathode at (a) 1st, (b) 15th and (c) 40th cycles.

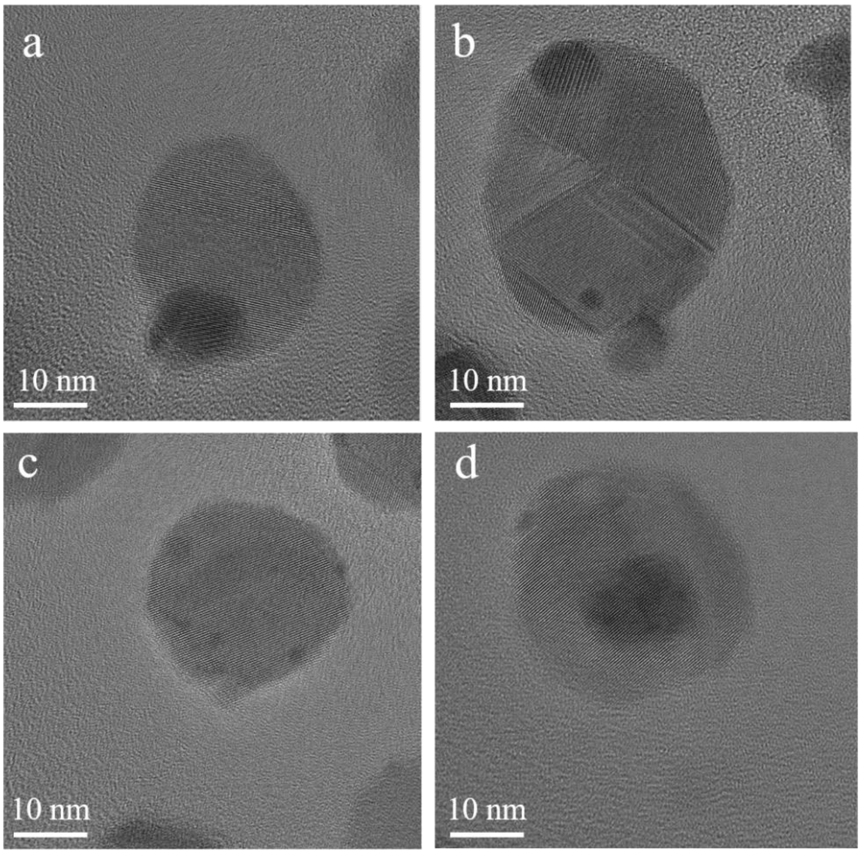

Figure S25. TEM images of the cycled H-Se cathode after 1 cycle.

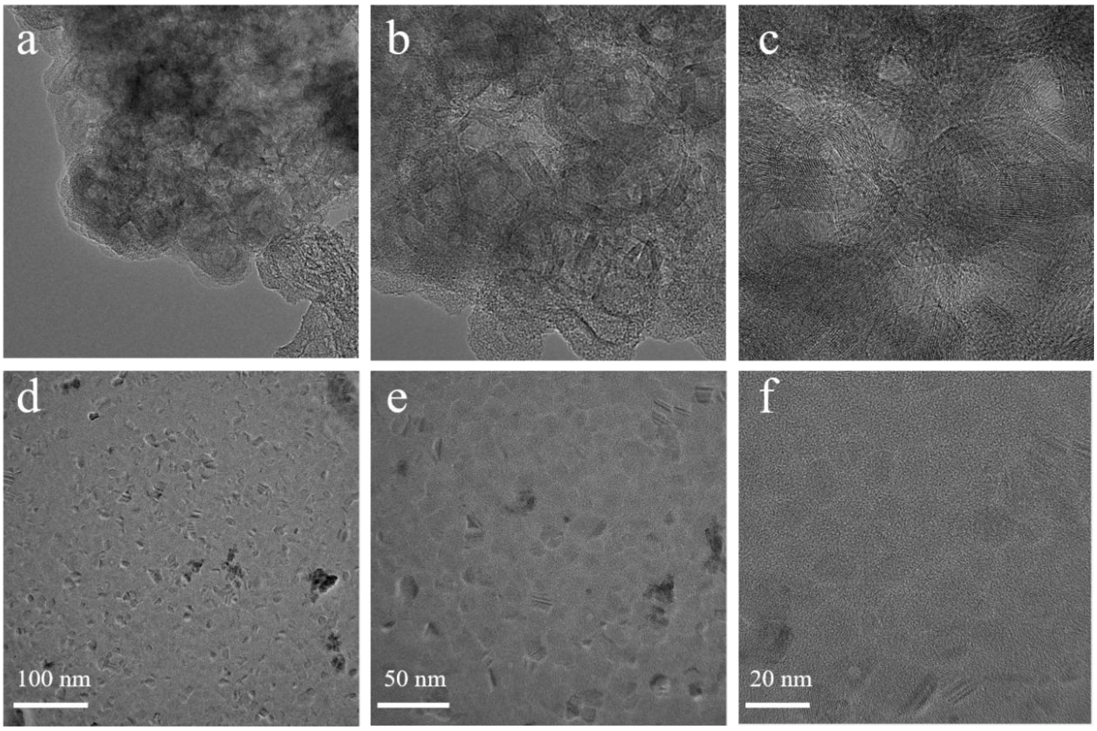

Figure S26. TEM images of the cycled M-Se cathode after 1 cycles.

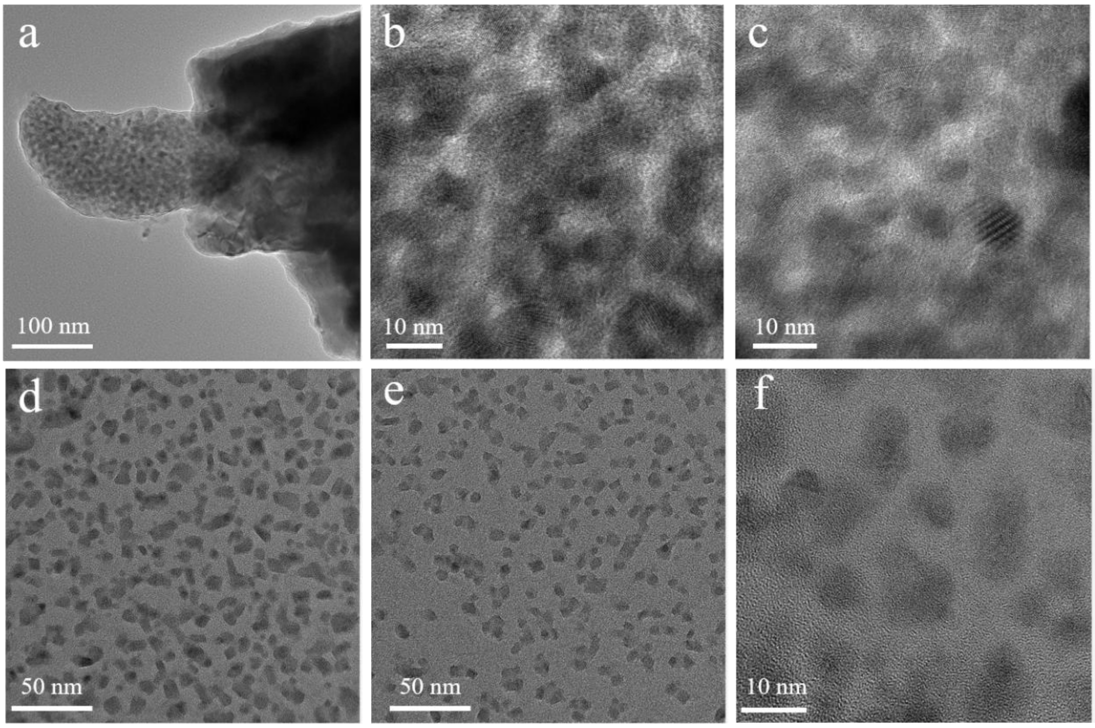

Figure S27. TEM images of the cycled A-Se cathode after 1 cycles.

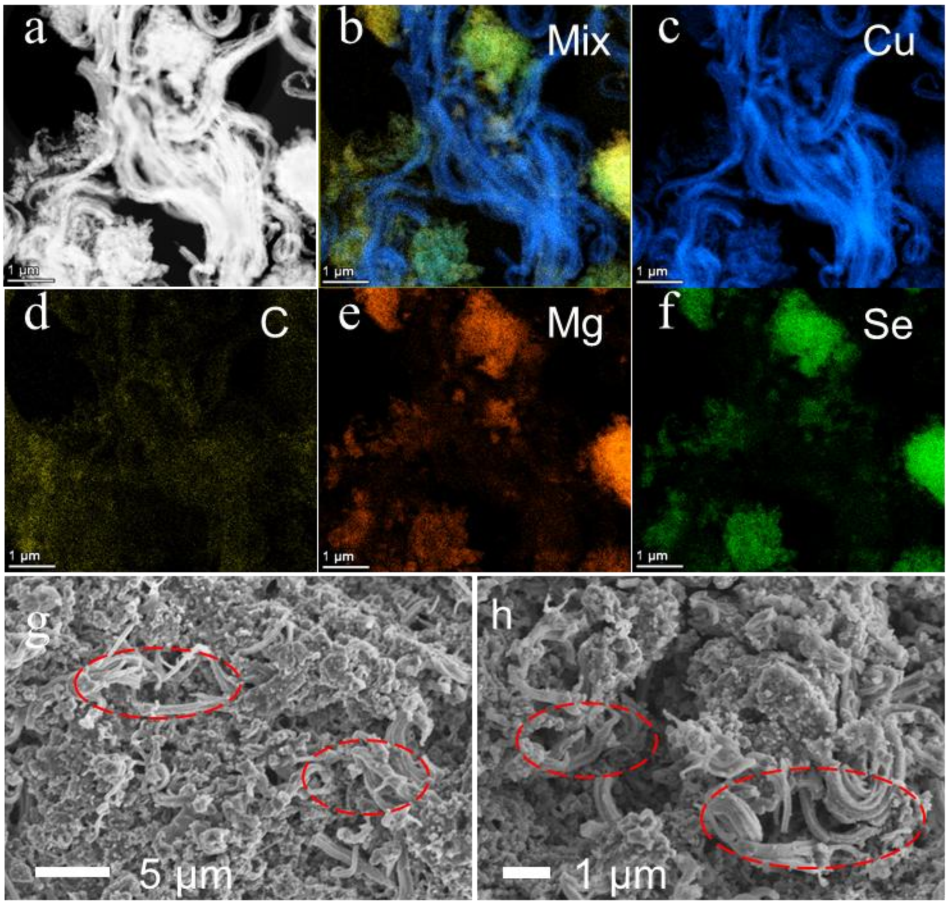

Figure S28. (a) STEM, (b-f) EDS mapping and (g, h) SEM images of the cycled H-Se cathode.

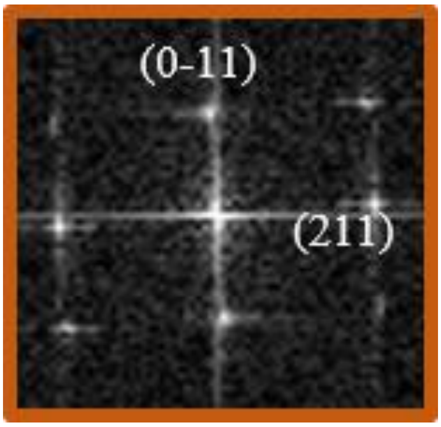

Figure S29. Diffraction patterns of the MgSe.

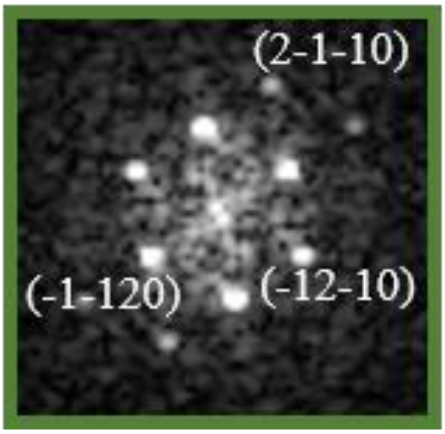

Figure S30. Diffraction patterns of the H-Se.

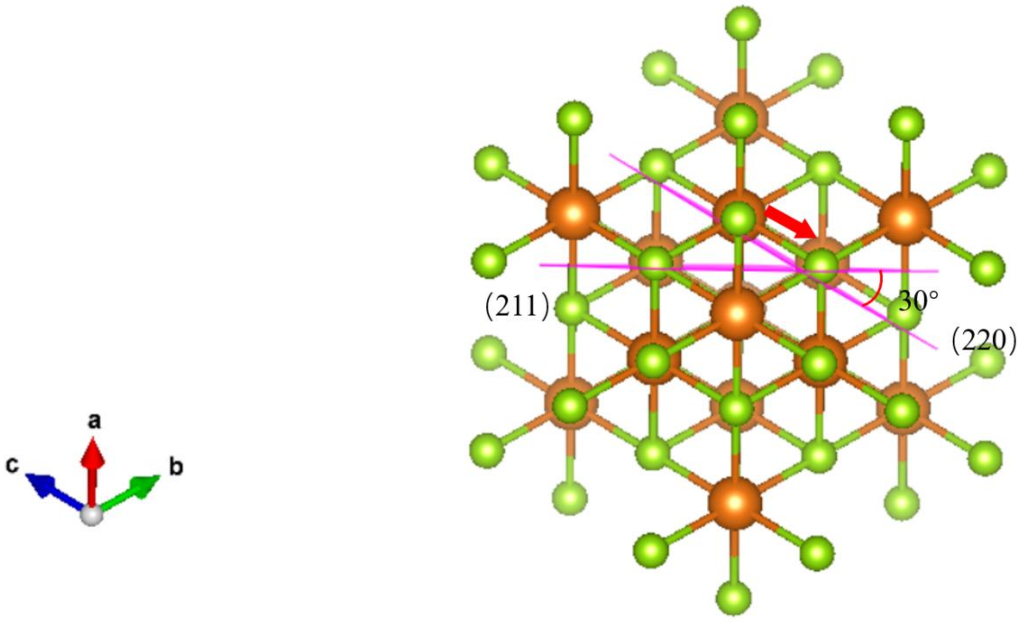

Figure S31. The angle between (211) and (220) planes of the cubic MgSe.

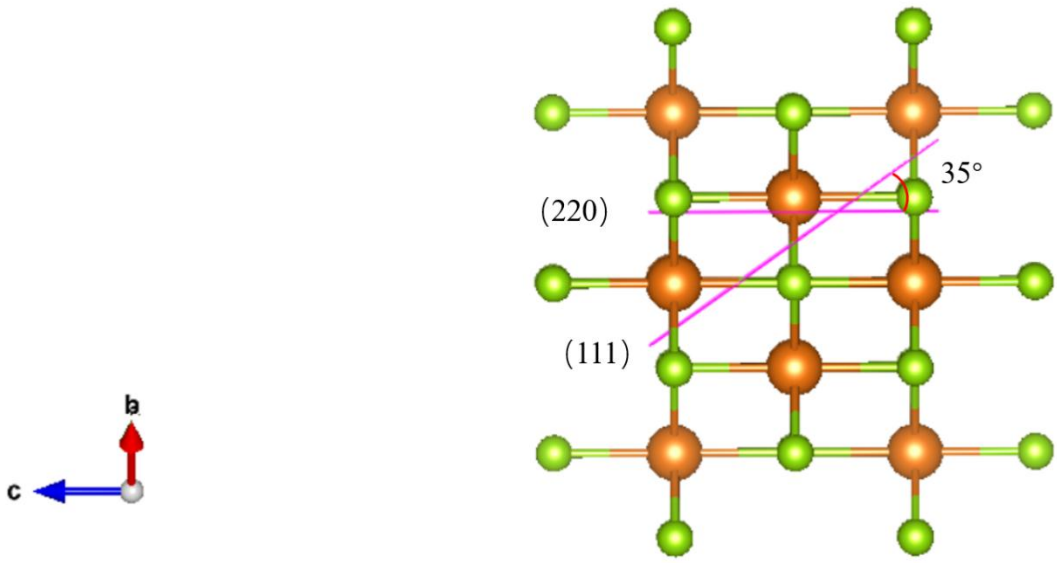

Figure S32. The angle between (111) and (220) planes of the cubic MgSe.

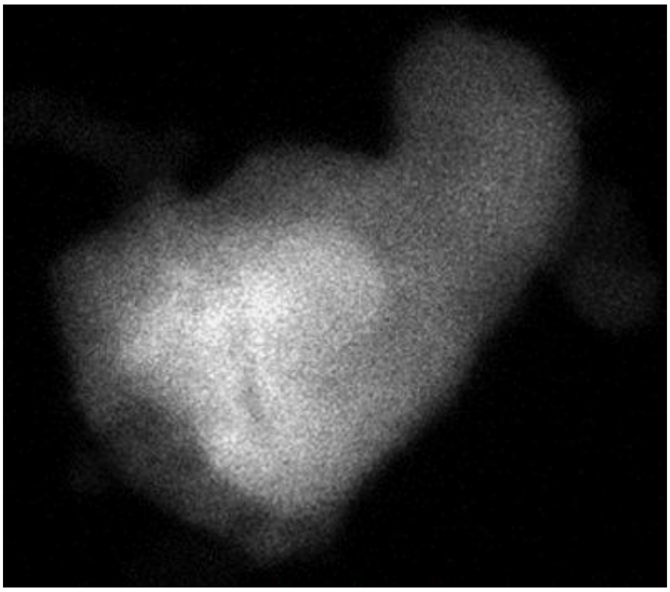

Figure S33. STEM images of the cycled H-Se cathode.

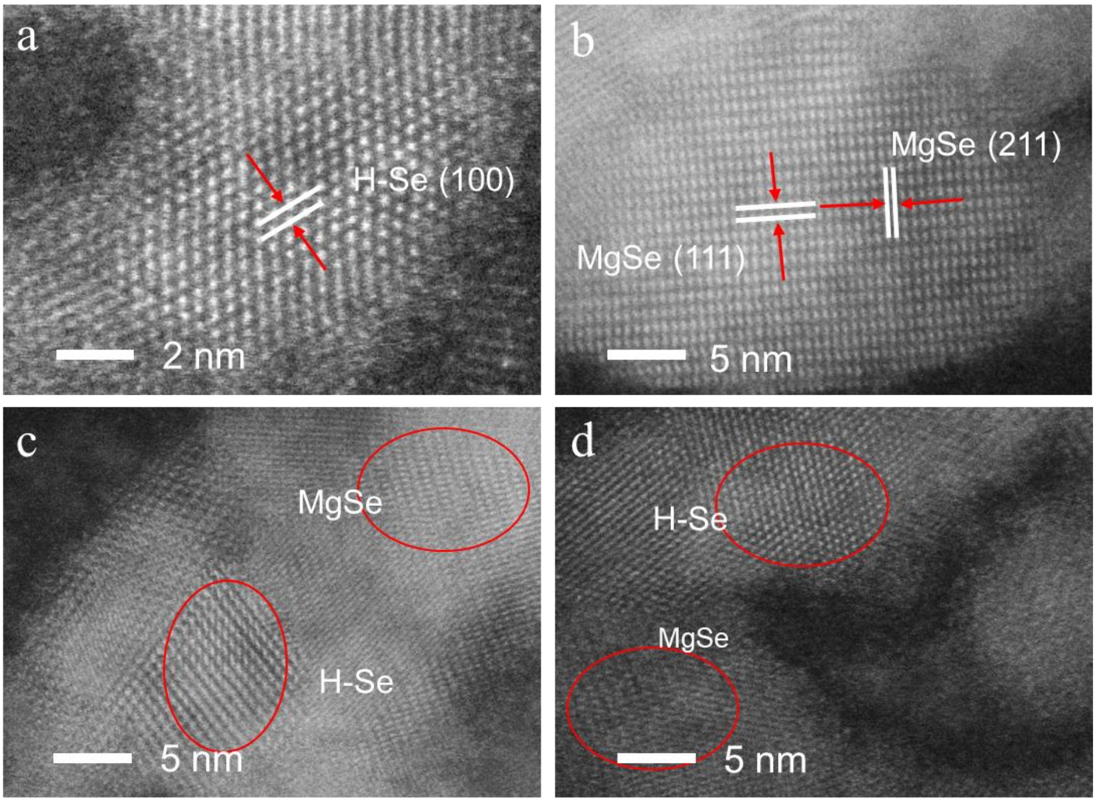

Figure S34. HADDF-STEM images of the cycled H-Se cathode with (a) H-Se, (b) $Cu_{2-x}Se$ and (c, d) H-Se and MgSe.

Table S1. Comparison of the electrochemical performance of different Mg electrolytes reported in the literature.

Cathode Materials	Energy density of cathode materials (Wh/kg)	Active Materials Loading (mg/cm^2)	Areal capacity (mAh/cm^2)	Energy density of total cathode and anode (Wh/kg)	Ref.
Mo6S8	80.5	11.8	0.708	48.4	1
PTO	630	2.5	0.75	127	2
S-R.T.	299	3	0.64	127	3
S-60	662	3.6	2.35	256.9	4
VS_4	390	2.8	0.84	140	5
PANI	320	2.5	0.625	100	6
$Mg_{0.15}MnO_2$	506	3	0.6	176	7
V_2O_5	149	0.5	0.01	22	8
FeS_2	605	2	1.2	172	9
This work	520	8	4.3	293	
This work	500	10	5.1	300	

Table S2. Performance comparison of H-Se with other cathodes.

Materials	Coin-cell Performance	Pouch-cell Performance	Reference
PTO	Specific capacity: 250 mAh/g<br>Areal capacity: 0.625 mAh/cm^2<br>Capacity retention: ~100%<br>Cycle number: 710	No data	2
$MgMnO_2$	Specific capacity: 182 mAh/g<br>Areal capacity: 0.6 mAh/cm^2<br>Capacity retention: 72%<br>Cycle number: 220	No data	7
S-60℃	Specific capacity: 904 mAh/g<br>Areal capacity: 1.18 mAh/cm^2<br>Capacity retention: 65.5%<br>Cycle number: 30	No data	4
CuS	No data	10 mAh	
S	No data	8.64 mAh	10
Mo_6S_8	No data	2.3 mAh	11
Mo_6S_8	No data	200 mAh<br>18 Wh/kg<br>(calculated)	1
H-Se	Specific capacity: 600 mAh/g<br>Areal capacity: 1.5 mAh/cm^2<br>Capacity retention: ~100%<br>Cycle number: ~1000	425 mAh<br>50 Wh/kg	This work

Table S3. Calculated energy density of Mg/Se pouch cell at different Se loading.

Pouch cell 1：

Parameter	Mg/Se in Fig. 1f
Active mass ratio	78%
Areal Se loading (mg/cm^2)	~1.6
Average discharge voltage (V vs. Mg^{2+}/Mg)	~1
Cathode (mg/cm^2)	~2
Cu foil (mg/cm^2)	9
E/S ratio	~3
Separator (mg/cm^2)	7
Mg foil (mg/cm^2)	8
Areal weight (mg/cm^2)	30.5
Total capacity (mAh)	67
Theoretical energy density (Wh/kg)	~31
Cycle number	>75

Pouch cell 2：

Parameter	Mg/Se in Fig. 1f
Active mass ratio	78%
Areal Se loading (mg/cm^2)	~3.2
Average discharge voltage (V vs. Mg^{2+}/Mg)	~1
Cathode (mg/cm^2)	~4
Cu foil (mg/cm^2)	9
E/S ratio	~2.5
Separator (mg/cm^2)	7
Mg foil (mg/cm^2)	8
Areal weight (mg/cm^2)	36
Total capacity (mAh)	90
Theoretical energy density (Wh/kg)	~51
Cycle number	20

Pouch cell 3：

Parameter	Mg/Se in Fig. 1f
Active mass ratio	78%
Areal Se loading (mg/cm^2)	~4.7
Average discharge voltage (V vs. Mg^{2+}/Mg)	~1
Cathode (mg/cm^2)	~6
Cu foil (mg/cm^2)	9
E/S ratio	~1.7
Separator (mg/cm^2)	7
Mg foil (mg/cm^2)	8
Areal weight (mg/cm^2)	38
Total capacity (mAh)	430
Theoretical energy density (Wh/kg)	~65
Cycle number	3

Reference：

1. J. A. Blázquez, R. R. Maça, O. Leonet, E. Azaceta, A. Mukherjee, Z. Zhao-Karger, Z. Li, A. Kovalevsky, A. Fernández-Barquín, A. R. Mainar, P.

Jankowski, L. Rademacher, S. Dey, S. E. Dutton, C. P. Grey, J. Drews, J. Häcker, T. Danner, A. Latz, D. Sotta, M. R. Palacin, J.-F. Martin, J. M. G. Lastra, M. Fichtner, S. Kundu, A. Kraytsberg, Y. Ein-Eli, M. Noked, D. Aurbach, *Energy Environ. Sci.* 2023, **16**, 1964.

2. H. Dong, O. Tutusaus, Y. Liang, Y. Zhang, Z. Lebens-Higgins, W. Yang, R. Mohtadi, Y. Yao, *Nat. Energy* 2020, **5**, 1043.
3. Z. Zhou, A. Du, W. Kong, Z. Chen, Z. Zhang, B. Chen, Y. He, S. Dong, Z. Li, G. Li, G. Cui, *J. Energy Chem.* 2022, **72**, 370.
4. S. Li, J. Zhang, S. Zhang, Q. Liu, H. Cheng, L. Fan, W. Zhang, X. Wang, Q. Wu, Y. Lu, *Nat. Energy* 2024, **9**, 285.
5. Z. Li, B. P. Vinayan, P. Jankowski, C. Njel, A. Roy, T. Vegge, J. Maibach, J. M. G. Lastra, M. Fichtner, Z. Zhao-Karger, *Angew. Chem. Int. Ed.* 2020, **59**, 11483.
6. C. Li, A. Shyamsunder, B. Key, Z. Yu, L. F. Nazar, *Joule* 2023, **7**, 2798.
7. S. Hou, X. Ji, K. Gaskell, P.-f. Wang, L. Wang, J. Xu, R. Sun, O. Borodin, C. Wang, *Science* 2021, **374**, 172.
8. S.-B. Son, T. Gao, S. P. Harvey, K. X. Steirer, A. Stokes, A. Norman, C. Wang, A. Cresce, K. Xu, C. Ban, *Nat. Chem*. 2018, **10**, 532.
9. Y. Shen, Q. Zhang, Y. Wang, L. Gu, X. Zhao, X. Shen, *Adv. Mater.* 2021, **33**, 2103881.
10. J. Bi, Z. Zhou, J. Li, B. Li, X. Sun, Y. Liu, K. Wang, G. Gao, Z. Du, W. Ai, W. Huang, *Angew. Chem. Int. Ed.* 2024, **63**, e202407770.
11. J. Bi, Y. Liu, Z. Du, K. Wang, W. Guan, H. Wu, W. Ai, W. Huang, *Adv. Mater.* 2024, **36**, 2309339.
